# Investigating the suitability of *in vitro* cell lines as models for the major subtypes of epithelial ovarian cancer

**DOI:** 10.3389/fcell.2023.1104514

**Published:** 2023-02-13

**Authors:** Aideen McCabe, Oza Zaheed, Simon Samuel McDade, Kellie Dean

**Affiliations:** ^1^ School of Biochemistry and Cell Biology, University College Cork, Cork, Ireland; ^2^ The SFI Centre for Research Training in Genomics Data Science, Galway, Ireland; ^3^ The Patrick G Johnston Centre for Cancer Research, Queen’s University Belfast, Belfast, Northern Ireland, United Kingdom

**Keywords:** Ovarian cancer, cell line, subtype classification, RNA-Seq–RNA sequencing, Non-negative matrix factorization (NMF), correlation

## Abstract

Epithelial ovarian cancer (EOC) is the most fatal gynaecological malignancy, accounting for over 200,000 deaths worldwide per year. EOC is a highly heterogeneous disease, classified into five major histological subtypes–high-grade serous (HGSOC), clear cell (CCOC), endometrioid (ENOC), mucinous (MOC) and low-grade serous (LGSOC) ovarian carcinomas. Classification of EOCs is clinically beneficial, as the various subtypes respond differently to chemotherapy and have distinct prognoses. Cell lines are often used as *in vitro* models for cancer, allowing researchers to explore pathophysiology in a relatively cheap and easy to manipulate system. However, most studies that make use of EOC cell lines fail to recognize the importance of subtype. Furthermore, the similarity of cell lines to their cognate primary tumors is often ignored. Identification of cell lines with high molecular similarity to primary tumors is needed in order to better guide pre-clinical EOC research and to improve development of targeted therapeutics and diagnostics for each distinctive subtype. This study aims to generate a reference dataset of cell lines representative of the major EOC subtypes. We found that non-negative matrix factorization (NMF) optimally clustered fifty-six cell lines into five groups, putatively corresponding to each of the five EOC subtypes. These clusters validated previous histological groupings, while also classifying other previously unannotated cell lines. We analysed the mutational and copy number landscapes of these lines to investigate whether they harboured the characteristic genomic alterations of each subtype. Finally we compared the gene expression profiles of cell lines with 93 primary tumor samples stratified by subtype, to identify lines with the highest molecular similarity to HGSOC, CCOC, ENOC, and MOC. In summary, we examined the molecular features of both EOC cell lines and primary tumors of multiple subtypes. We recommend a reference set of cell lines most suited to represent four different subtypes of EOC for both *in silico* and *in vitro* studies. We also identify lines displaying poor overall molecular similarity to EOC tumors, which we argue should be avoided in pre-clinical studies. Ultimately, our work emphasizes the importance of choosing suitable cell line models to maximise clinical relevance of experiments.

## Introduction

Ovarian cancer (OC) is the most deadly cancer of gynaecological origin, with over 300,000 new cases and 200,000 deaths occurring in 2020 (Sung et al., 2021). In the United States alone, OC accounts for approximately 20,000 new cases and 13,000 deaths per year (Siegel et al., 2022). Epithelial ovarian cancer (EOC) represents the majority of ovarian malignancies (Jayson et al., 2014) and is further classified into five major subtypes–high grade serous (HGSOC), clear cell (CCOC), endometrioid (ENOC), mucinous (MOC) and low grade serous (LGSOC) ovarian carcinomas (Prat et al., 2018). These subtypes differ in their genetic profiles, precursor lesions, response to therapy and clinical outcome (Table 1). For example, LGOSC, CCOC and MOC display poor responses to traditional platinum based chemotherapies, whereas ENOC and HGSOC display initial chemosensitivity (Sugiyama et al., 2000; Schmeler and Gershenson, 2008; Gershenson et al., 2009; Mackay et al., 2010; Kim et al., 2012; Prat et al., 2018; Lheureux et al., 2019a; Babaier and Ghatage, 2020). Furthermore, the lack of *BRCA1/2* mutations and hormone receptor (HR) deficiencies in non-HGSOC/high grade ENOC subtypes limits the use of poly (ADP-ribose) polymerase (PARP) inhibitors in disease management (Lheureux et al., 2019a; Babaier and Ghatage, 2020). Targeted therapies aimed at exploiting subtype-specific mutations are currently being developed (Coward, et al., 2015; Lheureux et al., 2019a) but have yet to be widely applied clinically. As such, in order to develop novel and targeted therapies for HGSOC and non-HGSOC EOCs and to effectively study the pathogenesis of these heterogeneous subtypes, it follows that pre-clinical studies should make use of models that are both stratified by subtype and reflective of their respective primary tumors.

**TABLE 1:** Incidence, mutational landscape, precursor lesions, chemotherapy response, and prognoses of the five major subtypes of EOC.

	HGSOC	CCOC	ENOC	MOC	LGSOC	References
Incidence	70%	10%	10%	3%	<5%	Prat et al. (2018)
Commonly mutated genes	Near-ubiquitous *TP53* mutations	*ARID1A* most frequently mutated (50%–60% of cases)	*ARID1A* mutated in up to 30% of cases	*KRAS* and *NRAS* frequently mutated	*BRAF, KRAS* and *NRAS* commonly mutated	Shaw et al. (2002), Risch et al.(2006), Jones et al. (2010), The Cancer Genome Atlas Research Network, (2011), McAlpine et al. (2012), Emmanuel et al. (2014), Kanchi et al. (2014), Hunter et al. (2015), Friedlander et al. (2016), Itamochi et al. (2017), Maru et al. (2017), Murakami et al. (2017), Kim et al. (2018), Shibuya et al. (2018), Cybulska et al. (2019), Cheasley et al. (2019), Morice, et al. (2019), Gorringe et al. (2020), Pierson et al. (2020), Cheasley et al. (2022)
	*BRCA1/2* germline mutations or inactivation	*PIK3CA*, *KRAS*, *PTEN*, *SMARCA4* also mutated	*PIK3CA, KRAS, PTEN, CTNNB1* also mutated	Mutations in *ARID1A* and *PIK3CA*	*TP53* mutations rare	
		*TP53* mutations in up to 20% of cases	*TP53* mutations in ∼17% of cases	Amplification of *ERBB2* and copy number loss of *CDKN2A*		
				*TP53* mutations in up to 64% of cases, often confined to later stages		
Precursor lesions	Serous tubal intraepithelial carcinoma (STIC) lesions in the fallopian tube	Endometriosis and retrograde menstruation, adenofibroma	Endometriosis and retrograde menstruation, adenofibroma	Mucinous benign cystadenomas and mucinous borderline ovarian tumors (MBTs)	Serous borderline tumors (SBTs) in 60%–80% of cases	Lee et al. (2007), Medeiros et al. (2006), Kurman and Shih, (2010), Wiegand et al. (2010), Erickson et al. (2013), Ahn et al. (2016), Prat et al. (2018), Cheasley et al. (2019), Shih et al. (2021)
Response to chemotherapy	Initially responsive (80%) but 20%–30% of tumors relapse 6 months after treatment and can develop resistance	Poor response (15%) and resistance to platinum chemotherapy noted	High grade ENOCs initially sensitive to platinum chemotherapy	Intermediate response (15%–60%) and resistance to platinum chemotherapy noted	Poor response (26%–28%)	Sugiyama et al. (2000), Gershenson et al. (2009), Alexandre et al. (2010), Berns and Bowtell, (2012), Kim et al. (2012), Prat et al. (2018), Lheureux et al. (2019a), Morice et al. (2019)
Clinical outcome	Poor survival rates due to overall later stage diagnosis and recurrent tumors after chemotherapy	Intermediate, with a greater risk of venous thromboembolism than other subtypes. Late stage CCOC has a poorer prognosis than late stage HGSOC	Low grade ENOC more prevalent and favourable, poorer outcomes in high grade ENOC cases	As 65%–80% are diagnosed at an early stage, survival is favourable, later stages have a poorer prognosis than late stage HGSOC	Most favourable outcomes of all subtypes, along with low-grade endometrioid carcinoma	Mackay et al. (2010), Berns and Bowtell, (2012), Peres et al. (2018), Prat et al. (2018), Ricciardi et al. (2018), Soyama et al. (2018), Morice et al. (2019)

Cell lines are often utilized to study cancer biology, as a cheaper and less time-consuming alternative to patient derived explants or animal models. There exists over 100 OC cell lines, with around 70 commercially available through the American Type Culture Collection (ATCC), European Collection of Authenticated Cell Cultures (ECACC), Rikagaku Kenkyusho (Institute of Physical and Chemical Research, RIKEN), Leibniz Institute DSMZ, CellBank Australia (CBA) and/or the Japanese Collection of Research Biosources Cell Bank (JCRB) (Beaufort et al., 2014; Ciucci et al., 2022). Cancer cell lines are relatively cheap, easy to maintain, and facilitate rapid results compared to more complex organoid, animal and tumor-on-a-chip platforms (Ciucci et al., 2022). However, the utility of cell lines in translational and pre-clinical research has been questioned. Studies in liver cancer (Chen et al., 2015), breast cancer (Jiang et al., 2016) and in particular, ovarian cancer (Anglesio et al., 2013; Domcke et al., 2013; Beaufort et al., 2014; Barnes et al., 2021) have demonstrated the limited capabilities of cell lines in accurately representing their corresponding tumor types. Importantly, these studies highlight that this poor applicability of models is exacerbated when histological subtype is not taken into account, emphasizing the importance of choosing suitable cell lines to maximize translation into the clinic. Indeed, a number of clinical trials have failed due to a lack of consideration for EOC subtype in pre-clinical studies (Coward et al., 2011) and the need for subtype-specific research in EOC to improve drug development and patient outcome has been emphasized (Alvarez et al., 2016; Lheureux et al., 2019b). This lack of subtype-specific research has been perpetuated by a lack of accurate cell line annotations, with the origin and subtype of most OC cell lines being poorly defined. Furthermore, the similarity of these models to their respective primary tumors is often not investigated or taken into account.

Since cell lines do not possess the morphological traits necessary for histological subtyping, current studies utilize molecular characteristics in order to determine the subtypes of these EOC models. A seminal study by Domcke et al. (2013) observed striking molecular differences between the most commonly cited cell lines and HGSOC tumor samples. By comparing copy number changes, mutational landscape and gene expression between 47 EOC cell lines and thousands of HGSOC tumor samples, the authors recommend a set of cell lines that best represent HGSOC primary tumors. Yu et al. (2019) also correlated molecular data of cell lines and tumors for 22 cancer types, including HGSOC, identifying the cell lines most correlated to their respective primary tumors. Efforts have also been made to identify CCOC cell lines (Anglesio et al., 2013). In this study, Anglesio and colleagues combined a panel of immunohistochemical biomarkers and a predictive algorithm to establish a subset of well-suited CCOC cell lines, as well as analyzing some HGSOC, ENOC and MOC models. Taking a wet-lab based approach, Beaufort et al. (2014) characterized 39 EOC cell lines in terms of gene expression and therapeutic response, partitioning cell lines in terms of both putative histological subtype and morphology. More recently, Barnes et al. (2021) expanded on the aforementioned work by utilizing non-negative matrix factorization (NMF) to separate 44 EOC cell lines into five groups that were suggested to correspond to all five EOC subtypes, expanding knowledge on cell line classification to other, non-HGSOC subtypes.

These studies have made remarkable progress in identifying the most suitable *in vitro* models to study different subtypes of ovarian cancer. However, there exists no study that compares the molecular profiles of EOC cell lines to primary tumor tissue of multiple EOC subtypes. Here, we integrate and analyse EOC cell line molecular data from multiple different sources, with the aim of generating a reference dataset of lines representative of the major EOC subtypes. We apply non-negative matrix factorisation (NMF) to a panel of 56 EOC cell lines. This resulted in five stable clusters that largely validated results obtained in previous studies, while also suggesting new subtype annotations for five previously unannotated cell lines. Additionally, genetic profiles of EOC cell lines were compared to 93 EOC primary tumor samples, from multiple independent datasets, stratified by subtype. We investigate the molecular similarity of cell lines to not only HGSOC primary tumors, but also CCOC, ENOC, and MOC. Ultimately, we identify the most and least representative cell lines to represent these EOC subtypes, generating a reference dataset for future *in silico* and *in vitro* studies. Consequently, our work highlights the need for the generation of additional, subtype specific datasets, in particular for the LGSOC and ENOC subtypes.

## Materials and methods

**FIGURE 1:**
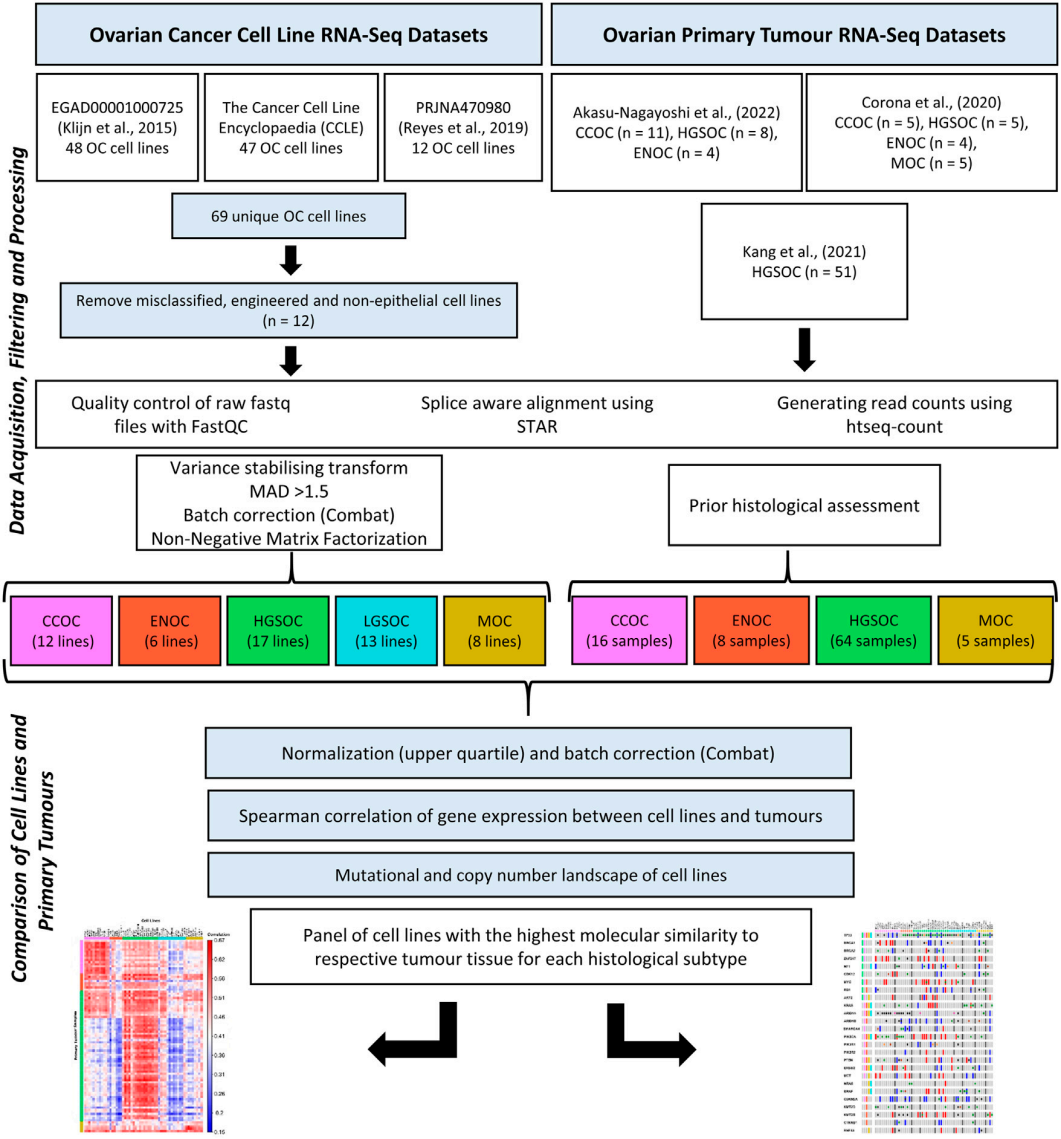
Transcriptomic data for 56 EOC cell lines and 93 primary tumor samples were processed using the same bioinformatics pipeline. NMF was used to stratify the cell lines into putative histological subtypes while patient samples had undergone histological assessment. Mutations and copy number variations of cell lines were investigated to identify the lines most similar to EOC tumor subtypes, as delineated in the literature. Correlation analysis was also carried out on the cell line and tumor gene expression data to assess similarity between the two groups.

An overview of the methodology followed is displayed in Figure 1.

### RNA-seq data acquisition and filtering

Ovarian cancer cell line RNA sequencing (RNA-seq) data was obtained from three sources: Klijn et al. (2015), the Cancer Cell Line Encyclopedia (CCLE, Ghandi et al., 2019) and Reyes et al. (2019). Raw sequence files from Klijn et al. (2015) were obtained with permission from the Genentech Data Access Committee (dataset ID EGAD00001000725) and downloaded from the EMBL-EGA servers using the pyEGA3 download client. For the CCLE and Reyes datasets, raw sequence files in FASTQ format were obtained from the European Nucleotide Archive (accessions PRJNA523380 and PRJNA470980). Klijn et al. (2015), CCLE and Reyes et al. (2019) provide RNA-seq data for 48, 47 and 12 OC cell lines respectively. In total, RNA-seq reads were available for 69 unique OC cell lines. An overview of datasets used and associated metadata are detailed in Supplementary Table S1.

OC cell lines that were misclassified, engineered or non-epithelial in origin were removed from analysis (*n* = 11, Supplementary Table S2). Two cell lines (DOV13 and OVCAR433) could not be processed due to errors with the EGA download client pyEGA3. 56 EOC cell lines remained for further analysis. A literature search on site of origin, original subtype annotation, *TP53* mutational status and treatment for each cell line and is detailed in Supplementary Table S3.

Ovarian primary tumor tissue data was obtained from three independent datasets (Corona et al., 2020; Kang et al., 2021; Akasu-Nagayoshi et al., 2022). Corona et al. (2020) provides both RNA-seq and chromatin immunoprecipitation sequencing (ChIP-seq) data on EOC tumors of various subtypes: CCOC (*n* = 5), HGSOC (*n* = 5), ENOC (*n* = 4) and MOC (*n* = 5). RNA-seq data for these 19 primary tumors were analyzed in this study. Kang et al. (2021) generated transcriptomic profiles for 51 HGSOC tumors of various stages and sensitivities to chemotherapy. Finally Akasu-Nagayoshi et al. (2022) conducted RNA-seq on stage III and IV primary tumor tissue of various subtypes: CCOC (*n* = 11), HGSOC (*n* = 8) and ENOC (*n* = 4). There is no publicly available RNA-seq data for LGSOC primary tumors (although a number of array-based sequencing datasets are available). In total, transcriptomic data for 16 CCOCs, 64 HGSOCs, 8 ENOCs and 5 MOCs were used.

### RNA-seq data processing

Quality control checks were performed on all FASTQ files using the FastQC package (Andrews, 2010). Forward and reverse reads for each cell line were mapped using the Spliced Transcripts Alignment to a Reference (STAR) software, version 2.7.9a (Dobin et al., 2013). The reads generated by Akasu-Nagayoshi et al. (2022), were single-end, which was taken into account during the alignment step. Reads were mapped to the human reference genome sequence (GRCh38) from Gencode (Frankish et al., 2021). Genome indices were generated using the comprehensive Gencode v39 gene annotation. Separate indices were required for each read length (Supplementary Table S1). Transcript level counts were generated using htseq-count (Anders et al., 2015). Strandedness of each dataset was taken into account during this step. Counts were compiled into a single matrix using R studio (v4.1.1). ComBat (Leek et al., 2012) was used to correct for batch effects between the three independent sources of data.

### Non-negative matrix factorization

**FIGURE 2:**
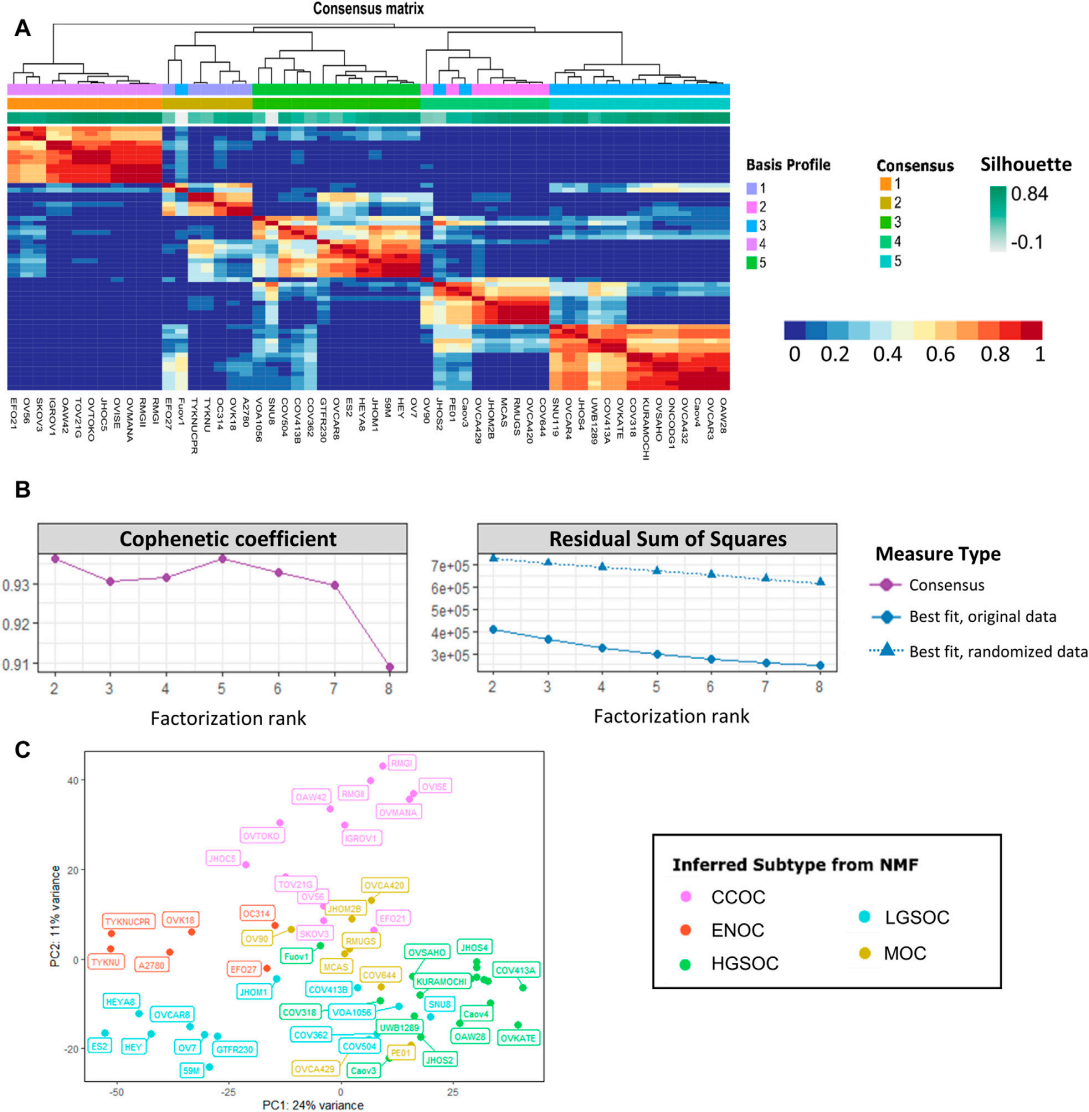
Non-negative matrix factorization separates 56 EOC cell lines into five stable clusters. **(A)** Consensus map detailing cell line clustering for 200 NMF runs using a factorization rank of 5. The colors of each heatmap tile represent the likelihood of two cell lines clustering together. Rows and columns of the map are symmetrically ordered by hierarchical clustering using the consensus matrix as a similarity measure. Above the heatmap are the associated dendogram, basis profile, consensus and silhouette scores. **(B)** Factorization rank survey on both original and permuted data shows high quality metrics for a five-group split. Left displays cophenetic correlation coefficient and right details the residual sum of squares for both the original and randomized data. Color and shape of points represent the type of measure used. **(C)** PCA plot of the 56 EOC cell line samples, colored by inferred NMF subtype, shows clustering of putative subtypes.

NMF was carried out on the processed count matrix, filtered to retain only cell lines, in R studio (v4.1.1). A variance stabilizing transform was applied to read counts using the DESeq2 package (Love et al., 2014). In order to retain the most variable genes, transcripts were filtered to exclude those with a median absolute deviation of less than 1.5. There were 2233 transcripts with median absolute deviation of ≥1.5; these were used as input for the NMF R package (Gaujoux and Seoighe, 2010). Estimation of factorization rank (*r*) was carried out by running NMF for values of *r* from 2 to 8, using 50 random initiation points. Quality measures including the cophenetic correlation coefficient, residual sum of squares and dispersion were used to select the most suitable value of *r*. In the presented data, clustering for *r* values of 2 and 5 displayed high quality metrics (Figure 2). After selection of a suitable *r* value, 200 runs of NMF were carried out with a factorization rank of 5 and a fixed random seed. Samples were assigned to one of five clusters based on maximum coefficient matrix values. The most likely subtype of each NMF cluster was inferred by comparison with previous studies and literature annotations.

### Mutational landscape of EOC cell lines and tumors

For cell lines present in the CCLE study, mutational and copy number landscape was originally determined by Ghandi et al. (2018) and visualized using cBioportal (Cerami et al., 2012; Gao et al., 2013). Mutational profiles of cell lines not present in the CCLE were determined from multiple sources (Anglesio et al., 2013; Beaufort et al., 2014; Tate et al., 2019). Copy number data was not available for 59M, UWB1289, OVCA420, OVCA432, PE01, HEY, RMGII, TYKNUCPR, COV413A, COV413B, GTFR230, VOA1056 or OVCA429. Mutational data was not available for a subset of genes in the RMGII, TYKNUCPR, COV413A, OVCA431, COV413B, GTFR230, VOA1056, and OVCA429 cell lines. A literature search was also performed to investigate the mutation frequency of 26 cancer driver/tumor suppressor genes in each of the five subtypes of EOC (Supplementary Table S4)

### Correlation analysis

The uniformly processed EOC cell line and tumor tissue counts were upper quartile normalized. ComBat (Leek et al., 2012) was then used to correct for batch effects between different sources of data. The normalized and batch corrected counts were filtered to keep the 2233 most variable transcripts in EOC cell lines (as identified earlier). The Spearman correlation was then calculated between cell lines and tumor tissues. Results were plotted as a correlation matrix using the corrplot package (Wei et al., 2021) and as boxplots using the ggplot2 package (Wickham, 2016), ordered by median correlation.

## Results

### NMF separates EOC cell lines into five clusters that reflect histological subtyping

Non-negative matrix factorization is a method of unsupervised learning often applied to gene expression data to extract biologically relevant information (Brunet et al., 2004; Gaujoux and Seoighe, 2010; Barnes et al., 2021). It functions by transforming a large, non-negative matrix (such as read counts) into a lower dimensional space: two smaller matrices *W* and *H*. The basis matrix *W* delineates the contribution of a small subset of genes to what are termed ‘metagenes’. This is essentially a decomposition of genes into those whose co-expression influences cluster assignment (Brunet et al., 2004). The coefficient matrix *H* details the co-expression pattern of metagenes in each sample, and can be used to cluster samples into a defined number of groups (*r*). Seeding is used to initialize the starting point for the NMF algorithm (i.e., to provide starting values for the basis and consensus matrices). The NMF package contains a number of built in seeding methods. Here the ‘random’ method is used, whereby these initial values are obtained from a uniform distribution with the same range as the input matrix. For reproducibility, a numerical value is passed into the seed function to seed the random number generator. In order to achieve a stable result from this random seeding method, multiple runs of NMF are required.

The number of metagenes and sample clusters are defined by the factorization rank (*r*), a critical parameter that is selected by the user-ideally, *r* should be small enough to reduce noise but large enough to preserve biologically relevant information. Brunet et al. (2004) suggests that the factorization rank should be chosen as the smallest value of *r* for which the cophenetic correlation coefficient starts decreasing. The cophenetic correlation coefficient indicates the robustness of clusters for a given choice of *r*. Frigyesi and Höglund (2008) suggest investigating methods other than the cophenetic correlation coefficient. It is argued that an increase in *r* is only relevant if the information captured by factorization is greater than that derived from permuted data, otherwise this increase will lead to overfitting. They suggest using the smallest value of *r* where the decrease in residual sum of squares (RSS) is lower than the decrease observed in randomized data. By comparing the residual error of NMF from the original data to that of permuted data, a solution is identified that is more information than noise, while preventing overfitting.

The consensus matrix also provides insight into the stability of the clusters. The entries of the consensus matrix reflect the probability two samples belong to the same cluster. The dispersion of the consensus matrix measures the reproducibility of clusters obtained from NMF; 1 for a perfect consensus matrix, and between 0 and 1 for a scattered consensus matrix. A consensus map can be plotted, which is a consensus matrix computed over multiple independent NMF runs, which is the average of the connectivity matrices of each separate run (Figure 2A). Quality of clustering can also be assessed using silhouette scores. This takes into account how similar a particular sample is to the other samples in its assigned cluster (mean intra-cluster distance), as well as the similarity to samples in other clusters (mean neighboring-cluster distance). A silhouette score close to 0 indicates the sample is on or near the decision boundary of two clusters whereas a silhouette score of 1 shows that the sample is distinct from clusters to which it does not belong. A negative silhouette score indicates a given sample has likely been erroneously assigned to a given cluster.

*r* was estimated by performing NMF with 50 runs of each value of *r* from 2 to 8. This was also completed for variance stabilized counts that had been permuted using the randomize function from the NMF package (Gaujoux and Seoighe, 2010). In the presented data, clustering for *r* values of 2 and 5 displayed high quality metrics (Figures 2A, B). Cophenetic correlation coefficients for *r* = 2 and 5 show robustness, and a drop in cophenetic correlation coefficient for *r* = 3 and *r* = 6 indicates less stability. For *r* = 6 the decrease in RSS in the observed data is less than the decrease in RSS in the randomized data. Therefore, a factorization rank of 5 was chosen for NMF analysis. The most likely subtype of each of the 5 NMF clusters was inferred by comparison with previous studies and literature annotations. In order to investigate the intra-cluster similarity, principal component analysis was carried out on the variance stabilized counts (Figure 2C). Each cluster assigned by NMF largely grouped together on the PCA plot. The ENOC and CCOC lines clustered away from the other samples, whereas there was some overlap between certain MOC, LGSOC and HGSOC cell lines. This suggested that some cell lines may have been misannotated by NMF or may display transcriptional characteristics of multiple EOC subtypes.

### NMF validates previous subtype classifications and assigns subtypes to five previously unannotated cell lines

The results obtained in using NMF analysis predominantly validated subtypes assigned in other studies (Supplementary Table S3). This study analyzed 43, 42, 22, and 20 EOC cell lines in common with Barnes et al. (2021), Domcke et al. (2013), Beaufort et al. (2014) and Anglesio et al. (2013), with subtype assignment agreeing in 39, 36, 15, and 14 cases respectively. Disagreements between our classifications and those of other studies added clarity to certain subtype assignments. We also utilized NMF to assign subtypes to five previously unannotated cell lines.

**FIGURE 3:**
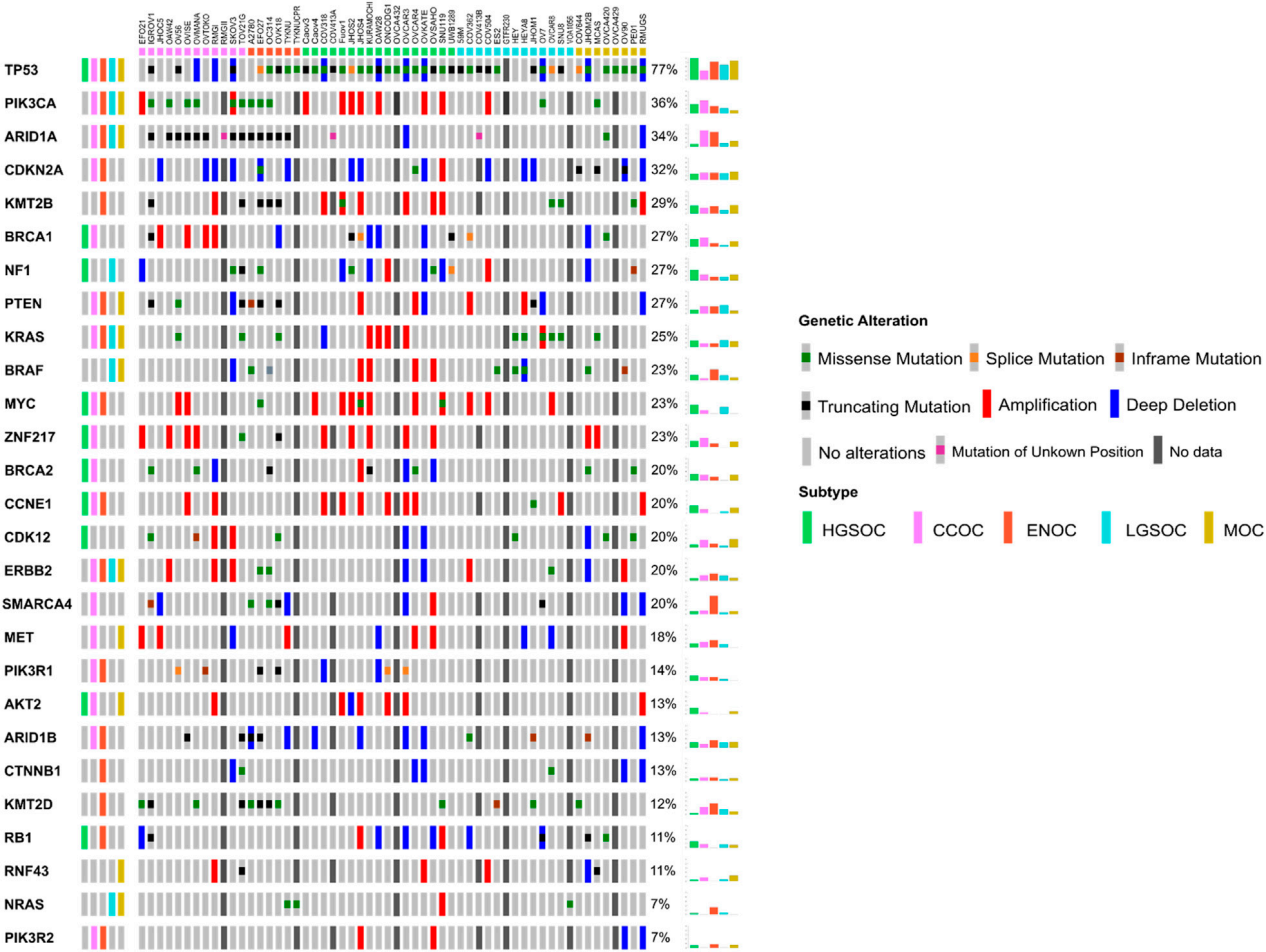
Oncoprint detailing the mutational and copy number landscape of 56 EOC cell lines in 27 genes commonly mutated in ovarian cancer, ordered by mutation frequency. Commonly mutated genes in each of the five subtypes were determined from a literature search (Supplementary Table S4) and shown in the leftmost column. Colored boxes on the top of the Oncoprint delineate the putative subtype of cell line samples as assigned by NMF. Mutation and/or copy number data was unavailable for a number of samples (see Materials and Methods) and are delineated with a dark grey box. Overall frequency of alteration/mutation for each gene in EOC cell lines by putative subtype are displayed in the rightmost columns.

There were a number of differences between the subtypes assigned to certain cell lines in this study compared to other previous works, often adding clarity to stratifications. OAW42, while classified as serous by Beaufort et al. (2014), was found to cluster with CCOC lines both here and by Barnes et al., (2021). Domcke et al. (2013) also classed this line as unlikely to be of HGSOC origin. The fact that OAW42 is *TP53* wild type, and possesses characteristic CCOC mutations (*ARID1A* and *PIK3CA,* Figure 3)*,* suggests that this line is likely a model of CCOC. OVCAR8 was classified as LGSOC both here and in Barnes et al. (2021), unlikely to be HGSOC by Domcke et al. (2013) and as HGSOC by Anglesio et al. (2013). Although this line has a TP53 mutation and MYC amplification (both of which are common to HGSOC), it also possesses *KRAS* and *ERRB2* mutations (Figure 3), suggesting it is more likely a model of LGSOC, in agreement with Barnes et al. (2021) and Domcke et al. (2013). HEY was classified here as LGSOC, which directly contradicted with Anglesio et al. (2013), where this line was determined to be of HGSOC origin. HEYA8 is another cell line analyzed, which was derived from the peritoneal cavity of mice injected with HEY cells (Supplementary Table S3). HEYA8 was categorized as a LGSOC line both here and by Barnes et al. (2021). It is likely that HEY is of LGSOC origin, as it possesses characteristic LGSOC mutations (*KRAS* and *BRAF,* Figure 3)*,* and it is the clonal ancestor of the HEYA8 cell line. Finally, OV56 was classified as LGSOC in Barnes et al. (2021), unlikely HGSOC in Domcke et al. (2013) and ENOC/CC in Beaufort et al. (2014). It is important to note that although classified as LGSOC by Barnes et al. (2021), the authors suggest that OV56 more likely represents CCOC, due to the presence *of KRAS, ARID1A, PIK3R1* and *PTEN* mutations (Figure 3). Indeed, we found this to be true in our analysis, with OV56 clustering with the CCOC cell lines.

Five cell lines (GTFR230, OVCA420, OVCA429, OVCA432, and TYKNUCPR) have not been investigated in terms of EOC subtype since their creation, with original annotation data unavailable for four of these lines (OVCA420, OVCA429, OVCA432, and TYKNUCPR). Additionally, these cell lines are rarely cited in literature and as such, mutational and copy number data was largely unavailable (Figure 3). TYKNUCPR is from a population of TYKNU cells exposed to cisplatin chemotherapy, so it is likely that these cell lines share a common subtype. Indeed, both lines were shown to cluster together, with putative ENOC cell lines. GTFR230 was originally derived from a stage IC MOC, although we show here that it clustered with LGSOC lines with a relatively high silhouette score (Figure 2A). As no mutational or copy number data was available for this line, an investigation into the similarity of GTFR230 to LGSOC and MOC tumors could not be carried out. This highlights the importance of other methods to determine subtype molecular similarity. OVCA432 was assigned as HGSOC in this study, while OVCA420 and OVCA429 both clustered with MOC lines. Mutational data was available for OVCA420, which showed that this line may possess characteristics of both HGSOC (*TP53, BRCA1, CDK12, RB1* mutations) and ENOC/CCOC/MOC (*ARID1A*) (Figure 3). Ultimately, we assign potential subtypes to these five cell lines based on NMF cluster membership. However, we note that due to the lack of additional mutational and copy number data, further investigation is needed to confirm classification of not only these lines, but all lines analyzed in this study.

### Integration of cell line molecular data highlights the most and least suitable models for subtype specific research

As discussed previously, subtype assignment of certain cell lines can prove difficult to resolve using transcriptional data, especially when mutational or copy number data is unavailable. Comparison of cell lines to tumor tissue is required to determine the suitability of these *in vitro* models. Some progress has been made in identifying suitable cell lines to utilize as HGSOC models. Both Domcke et al. (2013) and Yu et al. (2019) compared various molecular characteristics of cell lines to primary tumors of HGSOC origin, providing researchers with recommendations of the most suitable HGSOC lines. As of yet however, no study has extended analysis of this nature to multiple subtypes, to compile a reference set of cell lines representative of other EOC subtypes.

**FIGURE 4:**
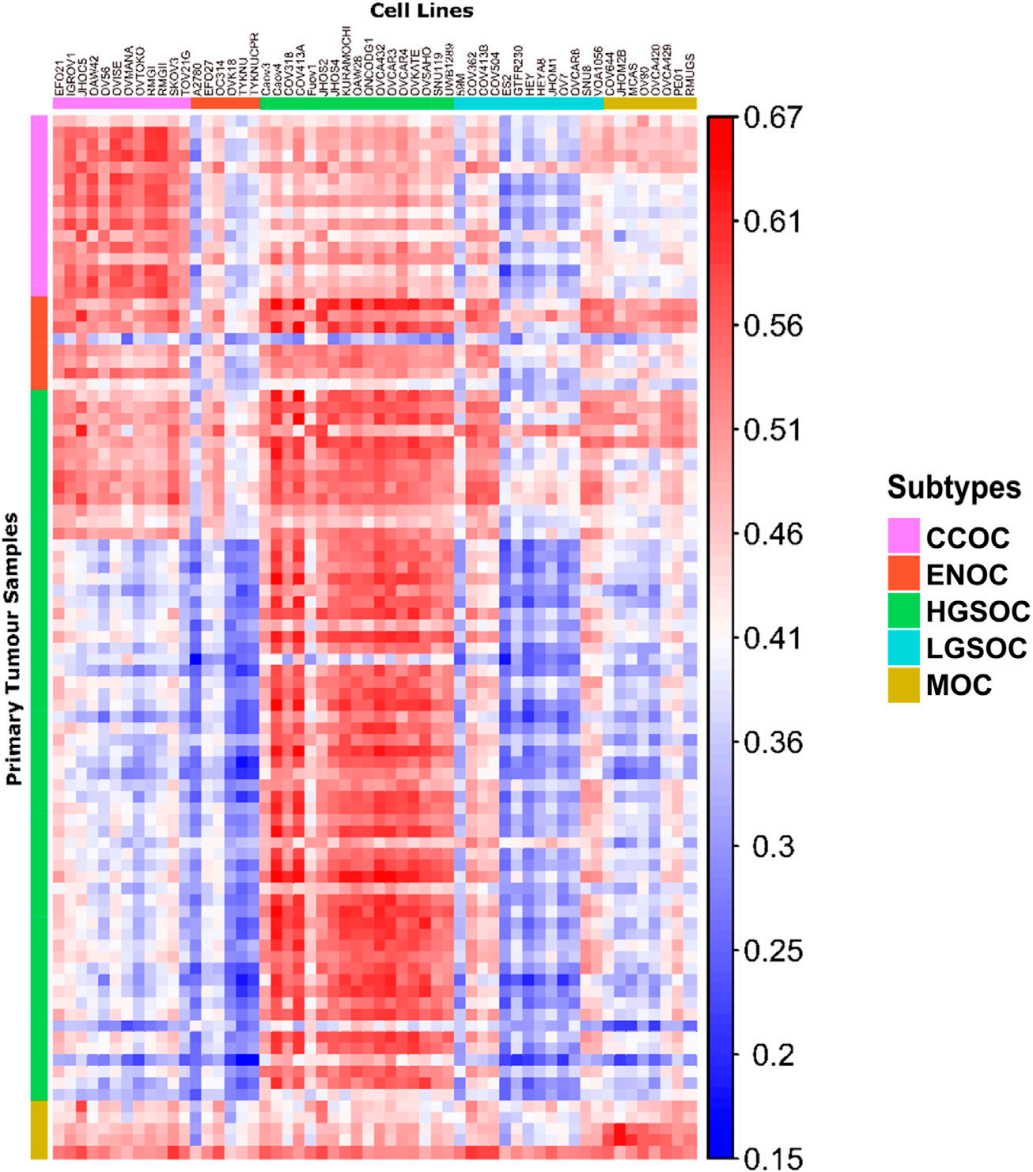
Correlation matrix comparing similarity of cell line models to primary tumor tissue of each subtype. Each column represents a cell line, with the putative subtype assigned by NMF delineated by colored boxes (top). Each row represents a tumor sample, with the subtype of samples as identified by histological assessment shown by colored boxes (left). Each tile shows the correlation of gene expression between a cell line and tumor sample, ranging from 0.15 to 0.67 (blue to red).

**FIGURE 5:**
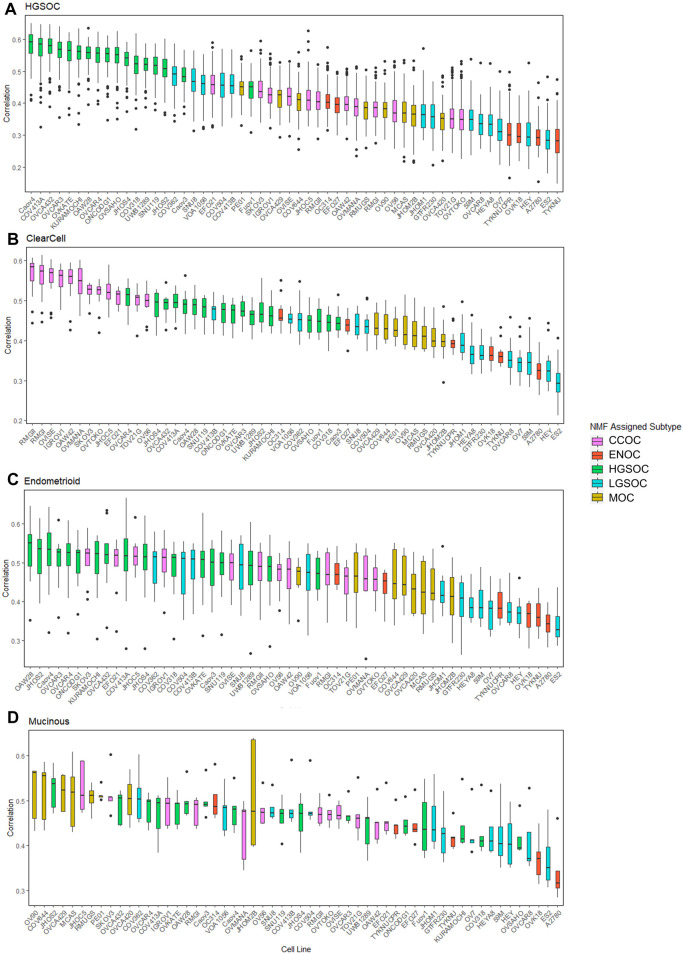
Distribution of correlations between cell lines and primary tumors of varying subtypes. Each boxplot is ordered in decreasing rank of median correlation between a particular cell line and all tumors of each subtype, **(A)** HGSOC tumors, **(B)** CCOC tumors, **(C)** ENOC tumors and **(D)** MOC tumors. Each box is colored based on subtype assigned to each cell line by NMF.

Publicly available transcriptomic data was available for primary tumors of HGSOC, CCOC, ENOC and MOC origins (93 samples in total, Supplementary Table S1). No RNA-seq data was available for LGSOC primary tumors, therefore this subtype could not be included in correlation analysis. Following normalization and batch corrections, we calculated correlation of gene expression profiles between EOC cell lines and primary EOC tumors (Figures 4, 5). Median correlations ranged from 0.28 to 0.59, with a similar range observed in Yu et al. (2019). In general, we observed that cell lines we assigned as HGSOC, CCOC and MOC were highly correlated to their respective primary tumor subtypes, whereas ENOC lines were poorly correlated to EOC overall (Figures 4, 5). We also note that most cell lines classified as LGSOC correlated poorly with tumors of HGSOC, CCOC, MOC and ENOC origin. Our data suggests that these lines may be useful models of LGSOC, although further analysis is needed to compare similarity to actual LGSOC tumor samples.

All HGSOC cell lines (apart from Fuov1) were within the top 20 most correlated cell lines to HGSOC primary tumors, with Caov4, COV413A, OVCA432, OVCAR3 and OVKATE representing the most highly correlated cell lines (Figure 5A). This study represents the first instance of subtype assignment of OVCA432, demonstrating its high similarity to HGSOC tumors. To validate that our ranking of HGSOC lines was consistent with both Domcke et al. (2013) and Yu et al. (2019), we first calculated the mean correlation of all cell lines with the 56 primary HGSOC tumors. We filtered this to include only the 39 cell lines analyzed by both Domcke et al. (2013) and Yu et al. (2019), and ranked cell lines in order of mean correlation with HGSOC tumors. Our suitability ranking was highly correlated with that observed by both Yu et al. (2019) (Spearman’s rho = 0.89, p-value <2.2e-16) and Domcke et al. (2013) order (Spearman’s rho = 0.58, p-value = 9.592e-05). A lower rho value was observed between our ranking, produced using gene expression data only, compared to that produced by Domcke et al. (2013), which took into account copy number alteration and mutational landscape. A higher rho value was observed between our ranking and that detailed in Yu et al. (2019), which was expected, as both studies also analyze transcriptomic data. However, in this study we analyze a completely independent set of 56 HGSOC tumors yet produce a similar suitability ranking.

Similar to HGSOC, cell lines classified here as CCOC possessed the highest ranking median correlations to CCOC tumors (Figure 5B). The 12 CCOC lines fell within the top 13 most correlated lines to CCOC tumors, with OVCAR4 (a HGSOC cell line) ranking 11th. The top 5 cell lines with highest mean correlation to CCOC tumors are RMGII, RMGI, OVISE, IGROV1 and OAW42. Although no mutational data is available for RMGII, its high correlation to CCOC tumors warrants its use as a CCOC model; however, mutational and copy number analysis on this line would be extremely useful to confirm this similarity. TOV21G and JHOC5 are also highly correlated to CCOC tumors, supporting recommendations for their use as CCOC models from Anglesio et al. (2013). It is important to note that Domcke et al. (2013) observed hypermutated genomes in TOV21G and IGROV1. Therefore, although these cell lines are highly correlated with CCOC tumors, caution should be taken when using these in experiments due to their hypermutated genomes. High correlation of OVCAR4 with CCOC tumors could be explained by the fact that this line harbors mutations that are seen in both HGOSC and CCOC (*BRCA2, MYC* amplifications), as well as those that are generally not observed in HGSOC (*MET* and *PTEN* amplifications, mutations in *CDKN2A*) (Figure 3). This high mutational overlap with CCOC may influence the high position of OVCAR4 in this ranking.

None of the six lines classified as ENOC ranked within the top 20 most correlated lines to ENOC tumors (Figure 5C). In fact, these cell lines were poorly correlated to tumors of HGSOC, CCOC and MOC. The most striking observation relates to A2780, a cell line reported to represent over 90% of citations in EOC studies (Domcke et al., 2013). A2780 has been previously reported to poorly model HGSOC tumors, and our results show that is a poor model of ENOC, the EOC tumor type it is expected to represent. Expression based clustering by Domcke et al. (2013) also showed that A2780 clusters closer to cancers of non-ovarian origin (such as lung, liver, stomach and small intestine) than to those of ovarian origin. Our analysis bolsters this observation, demonstrating that not only does A2780 display poor correlation with HGSOC tumors, but of EOC tumors of multiple subtypes. Based on our evidence, we recommend that use of A2780 for *in vitro* EOC studies should be avoided, and importantly, there is a striking need to develop additional ENOC cell lines. We observe a similar pattern for other lines classified as ENOC. Cell lines classified as HSGOC and CCOC may represent the best available models of this subtype, as they possess the highest median correlations to ENOC primary tumors (Figure 5C). Indeed HGSOC tumors have been noted to be highly similar to ENOC tumors of higher grades (Gilks et al., 2008; Karnezis et al., 2013; Lim et al., 2016) and CCOC tumors display a high mutational overlap with characteristic ENOC genomic alterations (Supplementary Table S4).

All lines classified as MOC (apart from JHOM2B) fell within the top 20 most highly correlated lines to MOC primary tumors (Figure 5D). COV644, MCAS, OVCA420, OVCA429, and RMUGS were amongst the lines with highest similarity. Again, this is the first reported instance of subtype assignment for OVCA420 and OVCA429, showing that these lines possess high molecular similarity to MOC tumors, despite a lack of mutational and copy number data. We also note here that GTFR230, while classified in this study as LGSOC, originated from a stage IC MOC tumor (Supplementary Table S2). We observed that this line ranks 48th (out of 56 lines) in terms of median correlation to MOC tumors, much lower than any putative MOC cell line (Figure 5D). We therefore suggest that this cell line is more representative of LGSOC, due to clustering with LGSOC lines. Further studies are needed to confirm this.

Most cell lines classified as LGSOC (HEY, HEYA8, JHOM1, OVCAR8, 59M, OV7, and ES2) were poorly correlated to primary tumors of HGSOC, CCOC, ENOC, and MOC origin (Figures 3–5). Additionally, these lines possess the major genomic alterations of LGSOC (Figure 3), and have been suggested to represent the LGSOC subtype by Barnes et al. (2021). We therefore hypothesize that these lines may potentially represent models of LGSOC. However, a comparison of these lines to primary LGSOC tumors is needed to confirm this, highlighting the need for additional datasets to be generated for this rarer EOC subtype.

**FIGURE 6:**
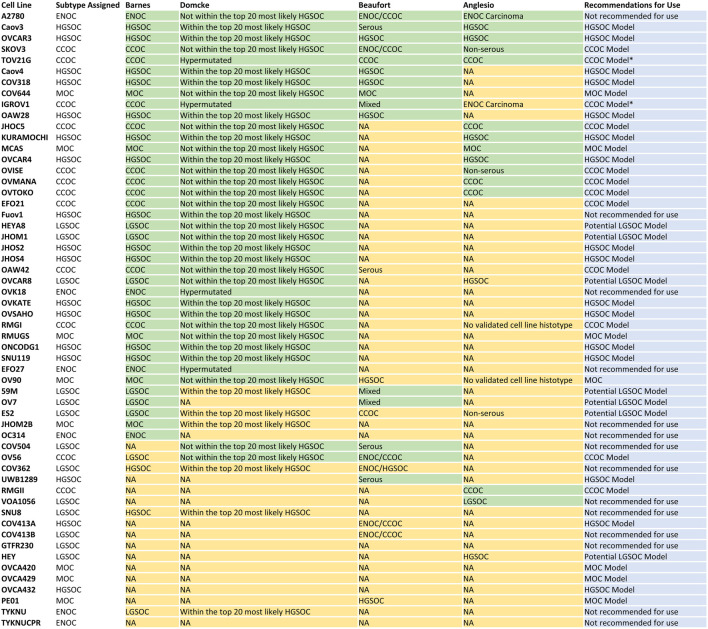
Consensus table displaying cell line subtype classification in this study, Barnes et al. (2021), Domcke et al. (2013), Beaufort et al. (2014) and Anglesio et al. (2013), and recommendations for future use. Overall, there are 16 models of HGSOC, 12 models of CCOC and seven models of MOC. Seven lines represent potential models of LGSOC, although further comparisons to tumor samples are needed to confirm this. Fourteen lines should be avoided in future *in vitro* and *in silico* studies due to i) poor correlation to EOC tumors overall (A2780, OVK18, OC314, EFO27, TYKNU, TYKNUCPR), ii) classification as a certain subtype but displaying poor correlation to respective primary tumors (Fuov1, JHOM2B), iii) high correlations to multiple EOC subtypes (SNU8), iv) classification as LGSOC but displaying high correlations to other EOC subtypes (COV504, VOA1056, COV413B, COV362) and GTFR230 due to origination from a MOC tumor but classification as LGSOC. *Caution should be taken when utilizing TOV21G and IGROV1 as models of CCOC due to their hyper mutated genomes.

Overall, we show that correlation of gene expression patterns between EOC cell lines and primary tumors can identify models with high molecular similarity to specific subtypes and aid in subtype identification, with the use of techniques such as NMF. In summary, we generated a reference dataset of cell lines most representative of HGSOC, CCOC, and MOC (Figure 6). We also highlight potential LGSOC models and those that are unsuitable for use in subtype specific studies.

## Discussion

In this study, we utilized publicly available transcriptomic, mutational and copy number data to investigate the molecular characteristics of 56 EOC cell lines. We demonstrated that cell lines optimally clustered into five stable groups that likely represent the five histological subtypes of EOC. We also compared the gene expression profiles of these cell lines to primary tumors of four EOC subtypes, generally observing that HGSOC, CCOC and MOC cell lines were highly correlated to their respective primary tumors. We noted that ENOC cell lines, especially A2780, were poorly correlated overall to not only ENOC tumors but EOC overall. We identified cell lines that feature the major genomic and transcriptomic features of each particular subtype and are therefore the most suitable *in vitro* models, at least for gene expression-based studies. Potential cell line models of LGSOC were also reported. Finally, we highlight the need for generation of additional subtype-specific datasets to help identify suitable models of less studied EOC subtypes, including LGSOC.

While this study is the first to correlate gene expression between EOC cells and primary tumor tissues stratified by multiple subtypes, it is not without its limitations. This study is purely an *in silico* investigation that lacks experimental validation. However, we note that our results are in agreement with other studies of this nature (Domcke et al., 2013; Yu et al., 2019) and are similar to other experimentally based studies of cell line subtype identification (Anglesio et al., 2013; Beaufort et al., 2014). Another potential limitation of this study is that we did not adjust for tumor purity in our correlation analysis, which has been identified as a confounder in previous studies (Yu et al. 2019). Finally, as our analysis was conducted using available genomic profiles, we did not include any LGSOC tumors. We also noted an overrepresentation of HGSOC tumor datasets compared to other subtypes. Since HGSOC is the most common and aggressive form of EOC (Prat et al., 2018), there is a disproportionate number of datasets representing this subtype, and comparably fewer examples of similar datasets for rarer, more indolent subtypes.

This evaluation on suitability of cell lines is by no means exhaustive, and is solely tailored to research questions involving gene expression. As our analysis was directed at correlation of transcriptomic profiles, we anticipate our results are most applicable in designing studies aimed at identifying biomarkers with elevated or reduced expression levels in certain subtypes of EOC. In general, determining the optimal tumor model depends on a myriad of considerations and circumstances. For example, future work should focus on identifying the most suitable set of cell lines for methylation or proteomic profiling studies, based on similarity with corresponding tumor genomic data. Additionally, the mutational status of genes other than BRCA1/2 included in clinical lab panels for genetic testing of heritable cancer syndromes could prove useful in identifying histotype-specific models. These include genes commonly mutated in Lynch Syndrome (*MLH1, MSH2, MSH6, PMS2,* and *EPCAM*), genes involved in homologous repair (*RAD51C, RAD51D, BRIP1*), or genes found to be associated with an increased risk of developing EOC overall (*STK11, CHEK2, PALB2, NBN, MRE11A,* and *RAD50*) (Harley et al., 2008; Li et al., 2019; Wagner et al., 2019; Amin et al., 2020).

It is possible that the use of two-dimensional, monocultures of individual cell lines may become greatly reduced or outdated in cancer research, due to the rising popularity of three dimensional *in vitro* models such as spheroids, multicellular organoid systems and tumor-on-a-chip models (Ciucci et al., 2022). Cell lines underrepresent the heterogeneity of tumors and the involvement of the tumor microenvironment, including other cells such as stromal cells (Wangsa et al., 2018, a colorectal cancer example). Cell lines also have higher somatic mutation rates than tumors, acquiring mutations through the culturing process (Kasai et al., 2016). In any case, there will be distinct disadvantages associated with any model system, and the ease of use and affordability of cell lines solidifies them as attractive model systems for lab researchers as starting points for drug screening or experimental optimization. Therefore, it is imperative to maximize the utility of these models, by selecting those that most accurately represent the tumor type to address the research question at hand.

In conclusion, although major technological progress and healthcare improvements have facilitated more effective cancer treatments, the last 20 years have only seen modest improvements in OC survival rates (Allemani et al., 2018; Arnold et al., 2019). Indeed, OC is a poor prognosis cancer, due in part to non-specific symptoms, a primarily imperceptible pre-invasive phase and development of resistance to traditional chemotherapies (Goff et al., 2004). Solutions to these problems have yet to be developed, owing to a lack of consideration for EOC subtypes in pre-clinical studies (Alvarez et al., 2016). In this work, we provide a potential reference for gene expression-based EOC studies to assist researchers in selecting appropriate cell lines to represent EOC subtypes. We concurrently highlight the need for more datasets, representing all subtypes to contribute towards the overall goal of expanding subtype-specific research in the field of EOC. A greater understanding of these diverse subtypes will no doubt lead to more targeted therapies, novel diagnostics and increased survival for patients affected by EOC.

## Data Availability

Publicly available datasets were analyzed in this study. This data can be found here: CCLE (https://www.ebi.ac.uk/ena/browser/view/PRJNA523380), Reyes et al. (2019) (https://www.ebi.ac.uk/ena/browser/view/PRJNA470980), Akasu-Nagayoshi et al. (2022) (https://www.ebi.ac.uk/ena/browser/view/PRJNA783540), Corona et al. (2020) (https://www.ebi.ac.uk/ena/browser/view/PRJNA495805), Kang et al. (2021) (https://www.ebi.ac.uk/ena/browser/view/PRJNA697947). Data from Klijn et al. (2015) was obtained with permission from the Genentech Data Access Committee (dataset ID EGAD00001000725). R scripts used in the NMF and correlation analyses can be found at https://github.com/aideenmc/FrontiersPaper_EOC_CellLines.
